# Assessment of Lower Limb Prosthesis through Wearable Sensors and Thermography

**DOI:** 10.3390/s140305041

**Published:** 2014-03-11

**Authors:** Andrea Giovanni Cutti, Paolo Perego, Marcello C. Fusca, Rinaldo Sacchetti, Giuseppe Andreoni

**Affiliations:** 1 Centro Protesi INAIL, Via Rabuina 14, Vigorso di Budrio (BO) 40054, Italy; E-Mails: ag.cutti@inail.it (A.G.C.); r.sacchetti@inail.it (R.S.); 2 Design Department, Politecnico di Milano, via Durando 38/A, Milan 20158, Italy; E-Mails: paolo.perego@polimi.it (P.P.); marcello.fusca@polimi.it (M.C.F.)

**Keywords:** gait, amputee, thermography, prosthesis assessment, wearable temperature and humidity sensors

## Abstract

This study aimed to explore the application of infrared thermography in combination with ambulatory wearable monitoring of temperature and relative humidity, to assess the residual limb-to-liner interface in lower-limb prosthesis users. Five male traumatic transtibial amputees were involved, who reported no problems or discomfort while wearing the prosthesis. A thermal imaging camera was used to measure superficial thermal distribution maps of the stump. A wearable system for recording the temperature and relative humidity in up to four anatomical points was developed, tested *in vitro* and integrated with the measurement set. The parallel application of an infrared camera and wearable sensors provided complementary information. Four main Regions of Interest were identified on the stump (inferior patella, lateral/medial epicondyles, tibial tuberosity), with good inter-subject repeatability. An average increase of 20% in hot areas (P < 0.05) is shown after walking compared to resting conditions. The sensors inside the cuff did not provoke any discomfort during recordings and provide an inside of the thermal exchanges while walking and recording the temperature increase (a regime value is ∼+1.1 ± 0.7 °C) and a more significant one (∼+4.1 ± 2.3%) in humidity because of the sweat produced. This study has also begun the development of a reference data set for optimal socket/liner-stump construction.

## 1. Introduction

The socket is the part of a lower-limb prosthesis that contains the residual limb and it is the medium that amputees use to control the artificial leg. Due to the variability in shape, bony and soft-tissue conditions of the stump, the socket is always designed, fabricated and tuned for each patient, starting from a plaster mold. This is a complex, time-consuming clinical and technical procedure and its outcome dictates the success of the prosthetic fitting to a large extent. To improve comfort and suspension of the prosthesis on the residual limb, components called “liners” have been introduced on the market. Liners are donned like a sock on the stump and as such are interposed between the limb and the socket. Liners, however, did not fully solve the issues regarding excessive mechanical stress produced by socket defects that generate painful areas. Moreover, they can still cause perspiration to accumulate between the residual limb and the liner and potentially cause, in combination with heat and friction, dermatologic problems [1,2].

Technologies and protocols that can assist the prosthetist in targeted socket adjustments and to compare the effect of different liners on a subject-specific basis, would contribute to patient satisfaction, mobility and health. We think that infrared thermography and wearable technologies for temperature and humidity assessment might serve the purpose.

Infrared thermography allows to measure in real-time the superficial temperature of a body/object by means of a dedicated camera. The possibility of reliably measuring a temperature over a wide area [3–6], non-invasively (contactless) and with good spatial resolution [7–10], allowed this technology to begin spreading as clinical tool [4,11–22]. The quantitative measure of the state of the residual limb perfusion which is revealed by thermal maps may provide important information about:

(1) Existing defects of the socket, that during walking translate into excessive forces and in turn to temperature increase;
(2) Skin inflammations, e.g., as the result of the liner leading to excessive humidity and heating;
(3) The classification and treatment of phantom limb pain.

To date the literature reports only a few studies on this topic. In particular, Kristen *et al*. [22] carried out a quantitative study to demonstrate phantom or stump pain by thermography, revealing the presence of typical thermal patterns. They found that: (a) in the presence of the stump pain, a real circulation disturbance was highlighted by a distinctly lower temperature in the stump head region in comparison with the reference group; (b) an asymmetrical temperature rise was shown in localized areas corresponding to a pressure point, an infection, or a locally painful spot; (c) phantom pain was mostly related to thermal maps presenting a patchy distribution of cooler areas directly around regions with relatively higher temperatures. A temperature decrease from the proximal part to the stump head was observed in all cases.

Wearable technologies can be applied to complement wide temperature maps with focused information on humidity inside the prosthesis during walking, integrated with temperature for a better assessment of the stump condition [1,2]. To the authors' knowledge, no literature is available on this regard.

Starting from these evidences and thanks to new miniaturized sensors, this study aimed at exploiting camera-based infrared thermography integrated with the ambulatory wearable monitoring of temperature (T) and relative humidity (RH) inside the prosthesis, for the assessment of the stump and of its interface with the liner. In particular, the system used for the assessment (hardware, software and measurement protocol), was expected: (1) to support in the analysis of temperature and humidity of the residual limb over time, e.g., before and after walking trials; and (2) to allow for the differential comparison of these parameters between measurement sessions. The goals of the present research were: (1) to develop and validate a wearable system measuring T and RH; (2) to propose an integrated clinical protocol based on infrared thermography and wearable sensors; and (3) to evaluate the *in-vivo* feasibility and relevance of this integrated protocol. Point 1 is addressed in Section 2, while points 2 and 3 are covered in Section 3. A general discussion and conclusions are reported in Sections 4 and 5, respectively.

## 2. Wearable System—Development and Validation

### 2.1. Materials and Methods

To collect temperature and humidity data, the SHT21S sensor produced by Sensirion (Staefa, Switzerland) was chosen due to its limited size (3 × 3 × 1.1 mm), resolution (0.04% RH and 0.01 °C) and expected accuracy tolerance (±2% RH, ±0.3 °C)—Table 1 [23]. Sensors were mounted on a 1 cm diameter miniboard. A datalogger was also implemented to record data from at most 4 sensors, concurrently. It was based on the Seeeduino Stalker board (Seeedstudio, Shenzhen, China) and incorporated four USB ports for sensor connection. Sensors were connected to the USB ports through flat 4-wire cables. The datalogger embedded a 2 Gb micro-SD memory card for data storage. The data logger was programmed to store one temperature and one humidity datapoint every 2 s. Before clinical application, sensors were tested *in vitro*, through comparison with a reference system (agreement analysis) in a controlled environment, to answer to three questions. Specifically, after sterilization of SHT21S sensors with sodium hypochlorite and subsequent reconditioning:

(1) Q1: What is the agreement with respect to the reference system?
(2) Q2: Can agreement be improved through a simple calibration involving bias compensation?
(3) Q3: Which is the smallest difference between measurements of two sensors that should be considered as a real difference?

These questions are relevant, since sensors must be sanitized between measurements on different subjects and sensors are, at present, too expensive to be disposable (about 60€).

For the experiments a set of four SHT21S sensors which underwent from 10 to 15 sterilization cycles with sodium hypochlorite (S1–S4) was considered.

An industrial oven (Binder FD240, Tuttlingen, Germany) with an insulated chamber was used, to ensure a homogeneous distribution of the temperature around the sensors. Temperature within the chamber can be set with a resolution of 1 °C. The actual temperature in the chamber is visible through a digital display with the resolution of 1 °C. The oven thermometer has the specifications reported in Table 1.

As further element of comparison for the temperature and as single comparison for humidity, an Amprobe TR300 System (Everett, WA, USA, which embeds temperature and humidity sensors as well as a datalogger) was used (Table 1). The TR300 was set to record a temperature and humidity sample every 2 s.

**Figure 1.**
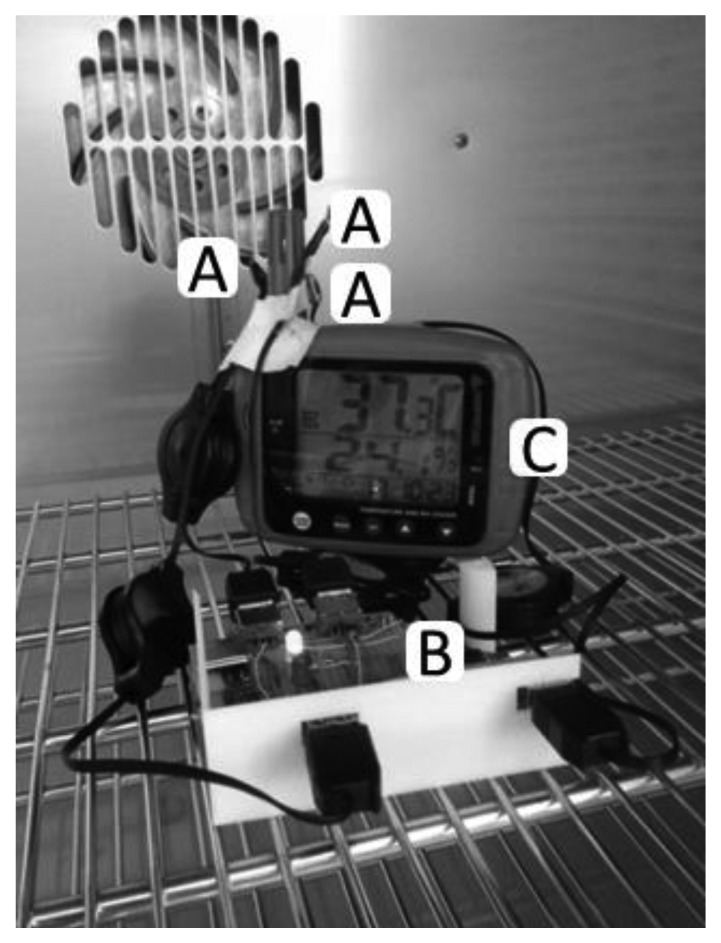
SHT21S sensors (**A**) connected to the Seeeduino Datalogger (**B**) and placed close to the sensing tip of the Amprobe TR300 (**C**) in the oven.

The testing procedure was as follows. After oven warm-up at 30 °C, the door was briefly opened to position all measurement systems at the center of the chamber, with the sensitive elements next to each other (Figure 1).

At the time of the TR300 first flash, indicating the start of the programmed recording, the Seeeduino Datalogger was activated and the oven doors were closed. The following temperature ramp was applied in steps of 5 min: 30°, 33°, 36°, 39°, 45°. The actual temperature readings and absolute time were noted from the FD240 display. Humidity could not be controlled, since the FD240 does not have this feature. After completion of the rump, the FD240 doors were opened and the systems stopped. Recordings were then downloaded from TR300 and the Seeeduino Logger to a personal computer.

**Figure 2.**
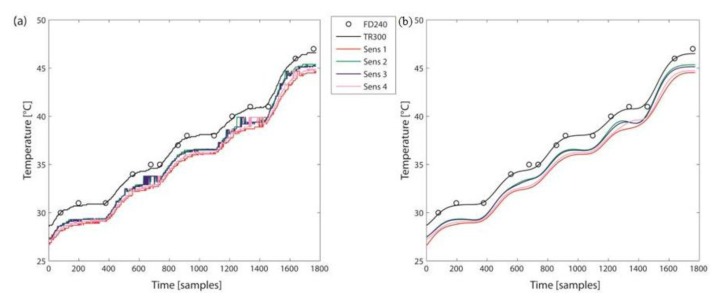
Example of (**a**) temperature raw data; and (**b**) temperature data after filtering.

Temperature and humidity recordings from all systems were overlapped for visual inspection (Figure 2a). Spectral analysis was then performed and the cut-off frequency for a Butterworth filter (4th order) was obtained. A low-pass Butterworth filter with cut-off frequency of 0.005 Hz was applied to all signals (Figure 2b).

Two sets of agreement analyses were then run:

- Agreement between TR300 and FD240: this analysis was intended to cross-check the agreement between the reference systems, to further confirm the use of TR300 as main reference, since TR300 records automatically with higher sampling frequency;
- Agreement between each of the “S” sensors and TR300: these four analyses provided the actual answer to Q1.

In particular, for each agreement analysis, a Bland-Altman plot was generated [24], reporting on the *x* axis the mean of the measurements of the two systems under analysis (true value), and on the *y* axis the difference between their measurements (error). Moreover, three quantities were computed:

(1) Root Mean Squared Error (RMSE), considering as input the measurements of the two instruments under comparison; this is a global parameter, that takes into consideration both the bias and the variability of the measurements;
(2) Bias: is the mean of the sample-by-sample difference between the measurements of the two instruments under consideration (as reported in the Bland-Altman plot);
(3) Coefficient of Repeatability (CR): 1.96 times the standard deviation of the sample-by-sample difference between the measurements of the systems under consideration; Bias ± CR defines the Upper and Lower Limit of Agreement of the Bland-Altman plot.

Generally, when there is no correlation between the error and the true value (X and Y in the Bland-Altman plot), a simple technique to recalibrate a sensor is the removal of the bias. On the contrary, when there is a correlation, the simple bias removal is ineffective. In these case, a model of the correlation between error and true value can be computed and then transformed to a calibration that applies to the original measures of the sensor in the time domain, so that:

(1) $$S_{i}^{\text{CAL}}(t)=S_{i}(t)\times m+q$$

where S^CAL^_i_ are the calibrated measures from the i-th Sensirion sensor, S_i_ are the original measures of the same sensors, *m* and *q* are the calibration parameters and *t* is the time.

To evaluate the effect of these simple calibrations (one or the other as appropriate) and answer to Q2, the RMSE was re-computed. To estimate the smallest difference between measurements of two SHT21S sensors that should be considered as a real difference, the Smallest Detectable Difference (SDD) among the four sensors was computed, based on Weir [25]. SDD calculation was repeated before and after calibration (Q2). SDD values were the base to answer to Q3.

### 2.2. Results

#### 2.2.1. Temperature

There was a good agreement between FD240 and TR300, as reported in Table 2. The RMSE = 0.3 °C even without the compensation for a limited offset. The CR was within 0.55 °C. These findings support the use of TR300 as reference for the Sensirion sensors.

**Figure 3.**
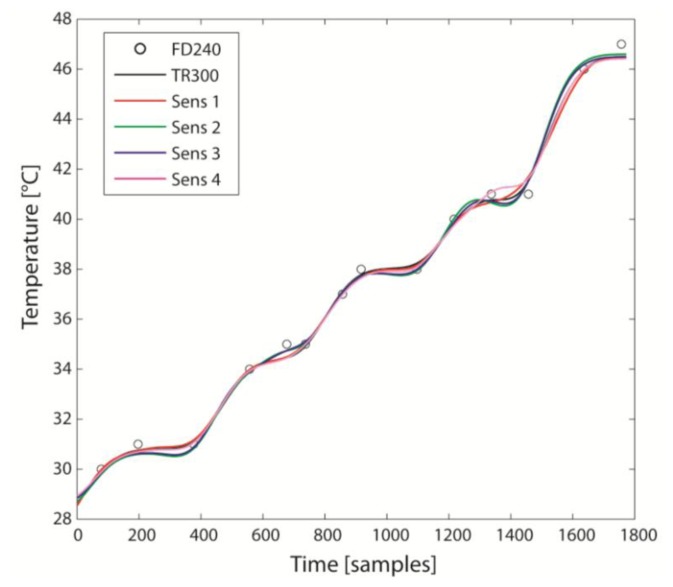
Temperature data after sensor-specific calibration (bias compensation). See Figure 2b for the same data before calibration.

The S sensors constantly underestimated the temperature compared to TR300, with agreement inferior to specifications (Tables 1 and 2), but with a small CR (<0.41 °C). The calibration, consisting in the sensor-specific compensation of the bias, considerably improved the agreement, with the maximum RMSE < 0.21 °C. After calibration, therefore, the agreement is just conditioned by CR and, at 95% probability, measurements from TR300 and one of the S sensors differ by ±0.41 °C. After calibration, the best sensor was #1 and the worst was #2, both within specifications (Table 2). The curve of temperature versus time after calibration is reported in Figure 3.

The analysis of the Smallest Detectable Difference (SDD) by the sensors points out a SDD_90_ = 0.91 °C with bias and 0.52 °C without bias. This means that differences between two sensors are real only if they exceed the SDD_90_. It is therefore important to compensate the bias to minimize the SDD.

#### 2.2.2. Relative Humidity

**Figure 4.**
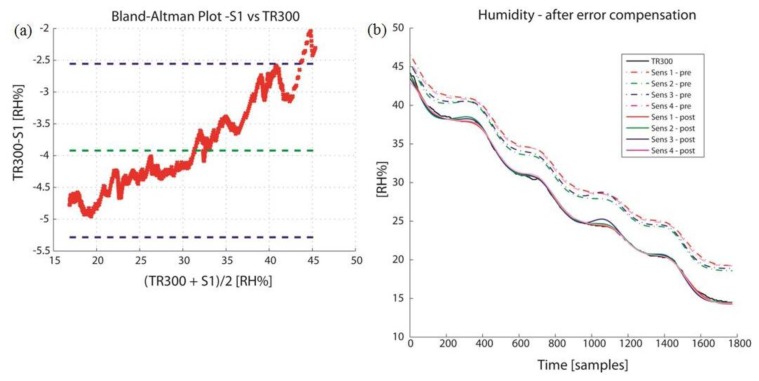
(**a**) Bland-Altman plot for S1 *vs*. TR300 for humidity. There is a clear correlation between the error (*y* axis, TR300-S1, [RH%]) and the true value estimate (*x* axis, 0.5 × (TR300 + S1), [RH%]); (**b**) Humidity signals from Sensirion sensors, pre- and post- calibration.

All the S sensors were consistent in overestimating the humidity compared to TR300, with 95% probability deviations, as reported by CR in Table 3. There was a clear correlation in the Bland-Altman plot between the error (Y) and the true value estimation (X) for all sensors, as reported in Figure 4a for Sensor 1. The application of a sensor-specific calibration model was effective in improving the agreement, (see Table 4 for the calibration model parameters in the time domain), with the final outcome reported in Figure 4b. All sensors are acceptable after calibrations, but Sensors 1 and 4 gave the best results.

The analysis of the SDD pointed out a SDD_90_ = 1.12% with bias and 0.51% without bias. This means that differences between two sensors are real only if they exceed the SDD_90_. Therefore sensors are in very good agreement both with and without bias compensation.

## 3. Clinical Protocol Based on Thermographic Imaging & Wearable Sensors

### 3.1. Materials and Methods

Based on the results reported in [20], infrared thermographic imaging might be able to identify the portions of the stump that are stressed by the socket. If reference maps were available, prosthetists would be able to discern between areas of normal *vs*. abnormal temperature (*i.e.*, stress) and thus rectify the socket accordingly. Similarly, the decision on the liner to use could be rationally based on the one leading to the lowest humidity during walking.

To these aims, a protocol was designed to measure temperature and humidity before and after walking, based on an infrared-camera and the wearable sensors described in Section 2. The aim if the present section is to describe the protocol and report an *in vivo* test to:

(1) Highlight consistent temperature patterns in transtibial amputees, before and after walking, e.g., similar spatial distribution of hot-spots;
(2) Verify if the wearable sensors SHT21S and connecting cables are painful for the amputee during walking.

**Figure 5.**
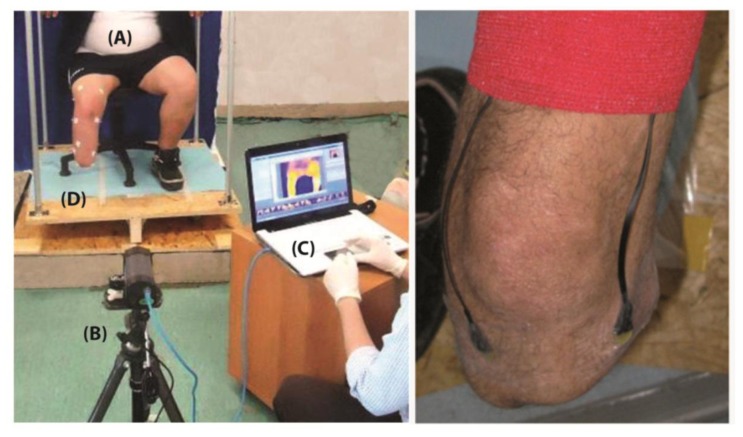
The experimental set-up (on the left): (**A**) the subject and his stump; (**B**) the IR camera for thermal map recording; (**C**) the PC for data processing; (**D**) a rotating platform for controlled image plane projections recording. The wearable sensor setup (on the right).

**Figure 6.**
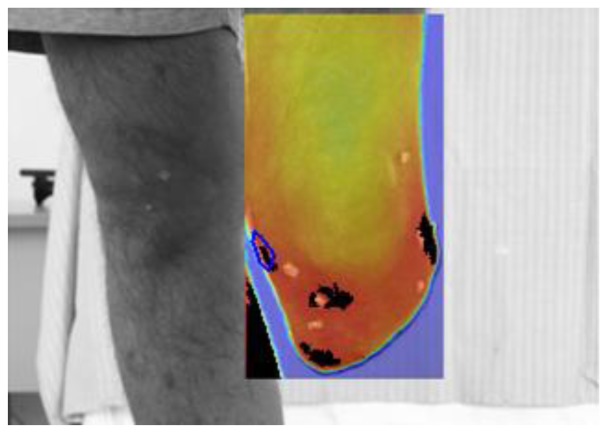
The image processing and the identification of the four ROI-hot spots at the human-prosthesis interface (on the right side, *i.e*., the stump of the left leg).

The protocol was implemented considering a FLIR A320 Thermal Imaging Camera (Temperature Range: −20 to 120 °C, image resolution: 320 × 240 pixels, Accuracy: 2%, Spectral range: 7.5 to 13 μm, Thermal sensitivity (NETD): <0.07 °C at 25 °C) for scanning body temperatures. Moreover, the system described in Section 2 was used, with welds connecting SHT21S to the 4-wire USB cable covered with silicone, to prevent skin irritation due to friction while walking. A standard digital camera is also required: we used a PowerShot A650 IS 12.1 Mpix digital camera (Canon, Tokyo, Japan). The experimental protocol consists of seven steps:

(1) Wearing the prosthesis, 15 min walking to stress the stump-prosthesis interface;
(2) Prosthesis removal (also the liner is removed) and immediate thermal maps and visible image recording (Figure 5);
(3) 15 min sitting for restoring basal conditions in a room with controlled temperature;
(4) “Basal-state” thermal map recording;
(5) Placement of the wearable sensors over critical areas identified during steps 1–4 (Figure 5);
(6) 5 min walking and sensors data recording;
(7) Sensors data download from the Seeeduino system and data processing.

In particular:

Regarding step 2, subjects are required to stand up on a spinning platform, with the stump vertical to the platform and aligned with its axis of rotation (Figure 5). Cameras stand on tripods and are not moved for collecting the different anatomical planes; instead the platform is rotated in steps of about 90° so that the whole subject rotates. While collecting basal images, the images collected after walking are used as reference to reposition the subject.

Regarding step 7, a dedicated software for infrared thermal maps analysis and integration with visible images and with the wearable sensors was developed in MATLAB (The Mathworks, Natick, MA, USA). The software implements a projective image-registration algorithm to superimpose “after-walking”, “basal” and visible images (Figure 6). The algorithm takes advantage of tiny ABS markers applied onto the subject's stump as starting guess for subsequent refinements. Then, isothermal images and differential thermal images are obtained for the whole image of specific regions of interest (ROI). The software also reads the Seeeduino data format and plots the time history of sensor recordings.

Five male subjects (mean age = 43.8 ± 6.2 years) participated to the study. They were traumatic trans-tibial amputees with time from the accident of 2 years or more; all of them reported no problems or discomfort while using the prosthesis. No restrictions in recruitment were applied according to prosthesis type. All subjects signed the informed consent prior to the recruitment to the study. For this preliminary test, sensors were placed lateral and medial to the tibial crest, distally (Figure 5). Moreover, two steps were added to the standard protocol:

(1) Infrared-images were collected after removing the wearable sensors, in the areas of their application, to check for signs of inflammation;
(2) Patients completed a VAS scale to indicate their level of discomfort while walking with the sensors in the prosthesis.

### 3.2. Results

The thermal imaging results are summarized in Table 5. An average increase of 20% in hot areas is shown after walking. This is consistent and statistically significant (paired t-test, P > 95%). These “possibly critical points” demonstrate good inter-subject repeatability: in 80% of subject they are identified in the inferior patella region and in the medial femoral epicondyle; two other areas are located in the tibial tuberosity area and on the lateral femoral epicondyle in 40% of subjects.

The SHT21 sensors did not provoke any discomfort to the patients during recordings and hot-spots in the areas of sensors application were not noticed (max VAS = 4). Information about interface temperature and humidity inside the cuff provided an inside vision of the thermal exchanges while walking. Sensors placed in the lower positions of the stump revealed a small increase in temperature (∼+1.1 ± 0.7 °C) and a more significant one (∼+4.1 ± 2.3%) in humidity because of the sweat produced. These results are consistent with the infrared imaging findings also in literature [20], with the additional possibility of recording the relative humidity.

## 4. Discussion

### 4.1. Discussion on Wearable Sensors

#### 4.1.1. Temperature

Based on the results reported in Section 2.2.1, we can formulate the following considerations and recommendations:

(1) Before compensation, agreement with TR300 is not fully acceptable;
(2) Agreement should be improved by sensor-specific bias compensation; this leads to an agreement (95%) of 0.41 °C, which is well within specification;
(3) The smallest detectable difference between Sensirion sensors is less than 1° (90% probability) when no bias compensation is performed and about 0.5° (90% probability) when it is compensated; calibration is then recommended.

#### 4.1.2. Relative Humidity

Based on the results reported in Section 2.2.2, we can formulate the following considerations and recommendations:

(1) Before calibration, agreement with TR300 is not acceptable;
(2) Agreement should be improved by sensor-specific calibration (bias compensation); this leads to an agreement (95%) of 0.77 °C, which is well within specification (Table 1); the model for bias compensation is reported in Table 4;
(3) The smallest detectable difference between Sensirion sensors is less than 1.12% (90% probability) before calibration and about 0.5% (90% probability) after calibration.

### 4.2. Discussion on Thermographic Imaging & Wearable Sensors—in vivo Test

The first aim of the study was the demonstration of the useful applicability of thermography in prosthetics. Furthermore we overcome the impossibility to measure the thermal parameters in dynamic conditions through the wearable sensors and data logger that non-intrusively records these data while the patient is freely walking. All recommendations reported in 4.1 were followed for the *in vivo* tests.

This study has begun the definition of a reference data set for trans-tibial amputee for optimal socket/liner-stump construction. Five main ROIs were identified on the stump with good inter-subject repeatability. Extension to a sample of five trans-femoral amputees is also ongoing: the preliminary results seems to confirm the reliability of the method and similar findings (with the logical obvious differences in hot spot positions).

## 5. Conclusions

In this study we exploited infrared thermography integrated with the ambulatory wearable monitoring of temperature and relative humidity in single body points inside the prosthesis for the assessment of the stump and of the interface with the liner.

Specifically we aimed to obtain specific information about:

(1) Agreement of wearable sensors for temperature and humidity measurements, formulating recommendations for their use;
(2) Information about humidity and temperature at the stumo-liner interface during walking;
(3) The presence of hot spots (and related parameters) at the liner-stump interface while walking and standing;
(4) A preliminary set of reference data and related statistics.

In accordance with the previous studies [3,19,22], our preliminary results suggest the possible use wearable technologies for these purpose, without any pain or discomfort for the amputee. Moreover, results suggest that thermography can highlight stressed areas at the stump-liner interface. Thermography appears as a promising screening test for the localization of inflammatory or painful areas, both during pre-prosthesis physical therapy and while wearing a prosthesis. In particular, thermography can support the study of the stump and the clinical decisions for:

(1) Adjusting the socket design;
(2) The localization of painful points to support the physician in the definition of analgesic therapy (e.g., laser therapy, capacitive-resistive-therapy);
(3) The localization of inflammatory situations supporting the clinician in the custom construction of the socket or in planning for a proper and localized anti-inflammatory therapy;
(4) The localization of thrombotic phenomena in amputees for peripheral vascular causes, in order to check the status of the limb before and after the prosthesis.

The parallel, the application of an infrared camera and wearable sensors provide complementary information. On one hand, a thermographic camera is non-invasive and collects thermal maps from wide areas of the stump, but is not suitable for real-time monitoring during gait. On the other, the wearable sensors collect information from very specific areas, but humidity can be measured as well, in combination with temperature.

## Figures and Tables

**Table 1.** Sensor specifications—accuracy.

	**Temperature**		**Humidity**	
	
	*Typical [*°*C]*	*Max [*°*C]*	*Typical [% ]*	*Max [% ]*
**SHT21S**	±0.3	±0.4	±2	±3
**Binder FD240**	±0.5	-	-	-
**Amprobe TR300**	±0.6	-	±3	-

**Table 2.** Outcome parameters for temperature measurements. CR is not affected by bias compensation, by definition. Sensors 1 and 4 are the best, Sensor 2 the worst. All measurements in °C.

**Sensor**	**RMSE (°C)**		**BIAS (°C)**		**CR**
		
	***Pre-cal***	***Post-cal***	***Pre-cal***	***Post-cal***	***Pre-cal/Post-cal***
TR300–FD240	0.30	0.28	−0.1	0	0.55
TR300–S1	1.94	0.10	1.94	0	0.20
TR300–S2	1.26	0.21	1.25	0	0.41
TR300–S3	1.36	0.17	1.34	0	0.33
TR300–S4	1.68	0.13	1.68	0	0.25

**Table 3.** Outcome parameters for humidity measurements. Since the compensation is on the error but translate into the original measures through a linear model, CR is also affected, so values are different pre- and post-calibration. Sensors 1 and 4 (underlined) are the best ones. All measurements in %.

	**RMSE (°C)**		**BIAS (°C)**		**CR**	
		
	***Pre-cal***	***Post-cal***	***Pre-cal***	***Post-cal***	***Pre-cal***	***Post-cal***
TR300–S1	4.00	0.53	−3.92	0	1.37	0.39
TR300–S2	3.08	0.60	−2.98	0	1.53	0.53
TR300–S3	3.38	0.39	−3.27	0	1.63	0.63
TR300–S4	3.80	0.37	−3.71	0	1.51	0.51

**Table 4.** Models for re-calibration of humidity: $S_{1,2,3,4}^{\text{CAL}}=S_{1,2,3,4}\times m+q$

	***S1***	***S2***	***S3***	***S4***
*m*	1.087	1.097	1.102	−1.095
*q*	−6.665	−5.940	−6.396	−6.688

**Table 5.** Hot spots and related inter-subjective average parameters.

**Walking**	**ROI1**	**ROI2**	**ROI3**	**ROI4**	**ROI5**	***MEAN***	***SD***
							
area (pixels)	443.33	325.50	218.00	1268.75	141.00	***479.32***	***455.77***
Tmax (°C)	44.04	32.54	32.36	33.38	32.80	***35.02***	***5.06***
Tmin (°C)	40.17	27.14	29.83	31.68	32.04	***32.17***	***4.88***
Tm (°C)	42.77	31.49	31.89	32.74	32.59	***34.30***	***4.77***
SDt (°C)	0.55	0.88	0.32	0.25	0.14	***0.43***	***0.29***
							
**Basal**	**ROI1**	**ROI2**	**ROI3**	**ROI4**	**ROI5**	***MEAN***	***SD***
							
area (pixels)	225.50	116.50	79.50	1275.00	246.00	***388.50***	***500.56***
Tmax (°C)	32.16	31.43	32.35	44.18	31.72	***34.37***	***5.50***
Tmin (°C)	31.25	28.08	31.68	41.41	31.00	***32.68***	***5.08***
Tm (°C)	31.79	30.87	32.08	43.33	31.44	***33.90***	***5.29***
SDt (°C)	0.19	0.46	0.15	0.24	0.13	***0.23***	***0.13***
							
**Positions**	**Medial condyle**	**Lateral condyle**	**Tibial tuberosity**	**Patella (below)**	**Head of the fibula**		
						
no.-walk	3	2	2	4	1		
no.-basal	4	2	2	3	1		
